# Entropic Approach to the Detection of Crucial Events

**DOI:** 10.3390/e21020178

**Published:** 2019-02-14

**Authors:** Garland Culbreth, Bruce J. West, Paolo Grigolini

**Affiliations:** 1 Center for Nonlinear Science, University of North Texas, P.O. Box 311427, Denton, TX 76203-1427, USA; 2 Department of Physics, Duke University, Durham, NC 27709, USA; 3 Information Science Directorate, US Army Research Office, Research Triangle Park, Durham, NC 27708, USA

**Keywords:** crucial events, renewal processes, ergodicity breakdown, stationary correlation function, renewal aging, heart-brain communication

## Abstract

In this paper, we establish a clear distinction between two processes yielding anomalous diffusion and 1/f noise. The first process is called Stationary Fractional Brownian Motion (SFBM) and is characterized by the use of stationary correlation functions. The second process rests on the action of crucial events generating ergodicity breakdown and aging effects. We refer to the latter as Aging Fractional Brownian Motion (AFBM). To settle the confusion between these different forms of Fractional Brownian Motion (FBM) we use an entropic approach properly updated to incorporate the recent advances of biology and psychology sciences on cognition. We show that although the joint action of crucial and non-crucial events may have the effect of making the crucial events virtually invisible, the entropic approach allows us to detect their action. The results of this paper lead us to the conclusion that the communication between the heart and the brain is accomplished by AFBM processes.

## 1. Introduction

In the last 30 years or so, the concept of anomalous diffusion has been widely adopted to deal with processes, ranging from biology to sociology, departing from the conditions of thermodynamic equilibrium that the Boltzmann principle [1] establishes for physical processes. For instance, in 1992, Peng et al. [2] introduced the concept of DNA walks that became a very popular way to study fluctuations of biological processes. They studied nucleotide sequences and assigned the symbol 1 to purines and the symbol −1 to pyrimidines. The position of a nucleotide is thought of as a time and the random walker at that time makes a step forward or backward according to whether the nucleotide is a purine or a pyrimidine. Since a nucleotide sequence is unique and it is not possible to adopt the conventional Gibbs prescription of making averages over many identical copies, the authors of this important paper adopted the method of a moving window of size *l*. The window moves along the nucleotide sequence and the observer records the space traveled by the walker in the “time” interval *l*, namely the distance from the position of the walker at time *l* to the position she had at the beginning of the window, assumed to be zero. In the case of random walk the fluctuations from 1 to −1 and back are totally uncorrelated and the resulting scaling η is equal to 0.5. These authors found that the scaling is larger than 0.5, thereby suggesting that the DNA nucleotides are correlated; or, said differently, the random walk is persistent.

What is the origin of this anomalous behavior? A widely shared conjecture is that the source of this correlation is properly described by means of the Fractional Brownian Motion (FBM) proposed by Mandelbrot [3]. This is a generalization of ordinary Brownian diffusion, yielding the following relation

(1) $$\frac{\langle x(-t)x(t)\rangle }{\langle x^{2}\left(t\right)\rangle }=1-2^{2H-1},$$

where *H* is the symbol adopted by Mandelbrot to denote scaling. Ordinary Brownian motion is a singularity of this formula, corresponding to H=0.5. This relation implies that FBM has memory of the infinitely distant past, since no limit is set on the magnitude of *t*. However, it has been noticed [4] that if we go beyond this mathematical formalism and adopt a dynamical derivation of FBM from the traditional diffusion equation

(2) $$\dot{x}\left(t\right)=\xi \left(t\right),$$

yielding in the integral

(3) $$x\left(t\right)=\int_{0}^{t}dt^{\prime }\xi \left(t^{\prime }\right),$$

we obtain the auto-correlation function

(4) $$\langle x\left(t_{1}\right)x\left(t_{2}\right)\rangle \equiv C\left(t_{1},t_{2}\right)\equiv \langle \xi^{2}\rangle \int_{0}^{t_{2}}dt_{2}^{\prime }\int_{0}^{t_{1}}dt_{1}^{\prime }\Phi_{\xi }\left(\right|t_{2}^{\prime }-t_{1}^{\prime }\left|\right),$$

where $\Phi_{\xi }\left(|t_{1}-t_{2}\right))$ is the stationary auto-correlation function $\langle \xi \left(t_{2}\right)\xi \left(t_{2}\right)\rangle /\langle \xi^{2}\rangle$. With a proper choice of this correlation function, setting $t\equiv t_{2}-t_{1}$ and sending *t*, $t_{1}$ and $t_{2}$ to ∞ has the effect of recovering Equation (1). For this reason, we define this form of infinite memory as Stationary Fractional Brownian Motion (SFBM).

Herein we adopt the symbol *H* to denote the SFBM scaling and the symbol η to denote the scaling of a differently described form of anomalous diffusion process.

The technological progress allowing the observation of the diffusion of single molecules in biological cells has attracted the general interest for a form of fractional diffusion that we term Aging Fractional Brownian Motion (AFBM) to stress its non-stationary nature [5]. The non-stationarity of this form of diffusion is not a consequence of the physical rules behind diffusion changing with time. However, rather, depends on the occurrence of crucial events that are responsible for the breakdown of ergodicity.

A good way to introduce the readers to crucial events is by adopting the engineering language of [6], which defines the age dependent failure rate g(t) through

(5) $$g\left(t\right)=\frac{\psi (t)}{\Psi (t)},$$

where Ψ(t) is the survival probability, namely the probability that a machine keeps working for a time interval *t* from the time at which it was created. Let us imagine that a team of engineers acts the moment a machine fails. They instantaneously correct the machine’s ill-functioning making it brand new. This has the effect of extending the working life of the machine to the next failure, when it will again require the instantaneous action of the team of engineers. The time distance between two consecutive failures has the waiting time distribution density

(6) $$\psi \left(t\right)=-\dot{\Psi }\left(t\right).$$

This leads to

(7) $$\Psi \left(t\right)=exp\left(-\int_{0}^{t}g\left(t^{\prime }\right)dt^{\prime }\right).$$

Assuming that g(t) decays in time as 1/t, namely at the limit of integrability, and more precisely according to

(8) $$g\left(t\right)=\frac{r_{0}}{1+r_{1}t},$$

when inserted into Equation (7) and integrating yields

(9) $$\Psi \left(t\right)={\left(\frac{T}{t+T}\right)}^{\mu -1},$$

where $T=1/r_{1}$ and $\mu =1+r_{0}/r_{1}$. Equation (9) readily yields the waiting time distribution density given by

(10) $$\psi \left(\tau \right)=\left(\mu -1\right)\frac{T^{\mu -1}}{{\left(\tau +T\right)}^{\mu }}.$$

We define the occurrence of these failures as the crucial events when the inverse power index μ fits the condition 1<μ<3. We note that the average waiting time, defined using Equation (10) is given by, when μ>2,

(11) $$\langle \tau \rangle =\frac{T}{\mu -2}.$$

To stress the non-stationary nature of this process, in the whole interval μ<3, as well as in the sub-interval μ<2, where the ergodicity breaking is made evident by the divergence of $\langle \tau \rangle$, let us assume that the laminar region between consecutive crucial events is filled with either 1 or −1, according to a coin tossing procedure. In this case, if we observe the process with the mobile window prescribed by the authors of Reference [2], with the constraint of locating the beginning of the window where we see an abrupt transition from 1 to −1 or from −1 to 1, we find

(12) $$\Phi \left(l\right)={\left(\frac{T}{l+T}\right)}^{\mu -1}.$$

We adopt the symbol Φ(l), rather than Ψ(τ), to stress the adoption of the mobile window to evaluate the brand-new survival probability. This is a consequence of the fact that when the window of length *l* overlaps more than one laminar region, different laminar regions may have opposite signs making the survival probability Φ(l) vanish.

If we do not adopt the above constraint and we move the left end of laminar region in a continuous way along the sequence, Φ(l) becomes the equilibrium correlation function defined by [7]:

(13) $$\Phi \left(l\right)=\frac{1}{\langle \tau \rangle }\int_{l}^{\infty }dl^{\prime }\left(l^{\prime }-l\right)\psi \left(l\right).$$

Using Equation (11) and inserting Equation (10) under the integral, direct integration yields

(14) $$\Phi \left(l\right)={\left(\frac{T}{l+T}\right)}^{\mu -2}.$$

This is a result of the non-stationary nature of this process that as effect of aging changes the power index μ of Equation (12) into μ−1. If we try to interpret this result by using SFBM we are immediately led to make the conjecture that the scaling index of Mandelbrot *H* fluctuates. In fact, the stationary correlation function $\Phi_{\xi }\left(t\right)$ yielding the surprisingly extended memory of Equation (1) has an inverse power law (IPL) tail with index δ related to *H* by

(15) δ=2−2H.

Consequently, if we identify the exponents of Equation (12) and Equation (14) with a non-stationary δ, we get the non-stationary *H* given by

(16) $$H=\frac{3}{2}-\frac{\mu }{2},$$

which, for instance, in the case μ=3, would change from H=0 (μ=3) to H=0.5 (μ=2).

The main purpose of the present paper is to propose an entropic approach to the analysis of time series that will establish if the anomalous diffusion emerging from the use of the mobile window of Reference [2] is due to the Mandelbrot infinite memory or to crucial events. We also posit suggestions to establish if both sources of anomalous diffusion are jointly acting on the complex process under study. We show that this entropic approach to the analysis of physiological data, on the dynamics of heart and the brain, settles the ambiguity about the 1/f noise generated by these physiological processes leading to the conclusion that they are driven by crucial events.

The outline of the present paper is as follows. In Section 2 we show that the observation of 1/f noise does not afford a clear-cut criterion to establish whether SFBM or AFBM applies. In Section 3 we illustrate the entropy concepts that are used in this paper to detect crucial events, by assessing if the experimental signal under analysis is a SFBM or an AFBM. Section 4 illustrates the main result of this paper, namely, how to prove that an anomalous scaling is a manifestation of crucial events. Section 5 affords detail on the method of stripes. In Section 6 we discuss the consequences of the main results of this paper for the dynamics of heart and the brain. Finally, in Section 7 we argue that the results of this paper go much beyond the limit of a single discipline and can be beneficial for psychology and sociology as well as for physiology and biology.

## 2. Pink Noise

In the literature there exists general consensus concerning the connection between criticality and 1/f noise (pink noise). In addition to the original work of Bak and coworkers [8], see also, for instance, the review paper [9] emphasizing the connection between criticality, 1/f noise and cognition. Here we show that 1/f noise can be derived from both SFBM and AFBM.

### 2.1. Stationary Fractional Brownian Motion

As pointed in Section 1, SFBM is usually referred to in the literature as FBM. The dynamical approach to FBM [4] yielding Equation (4), generates the following expression for the second moment of the diffusion process

(17) $$\langle x^{2}\left(t\right)\rangle =<\xi^{2}>\int_{0}^{t}dt^{\prime }\int_{0}^{t^{\prime }}dt^{\prime \prime }\Phi_{\xi }\left(t^{\prime \prime }\right).$$

According to Mandelbrot, the second moment scales in the following way, $\langle x{\left(t\right)}^{2}\rangle \propto t^{2H}$, thereby entailing the stationary correlation function $\Phi_{\xi }\left(t\right)$ to have, for H>0.5, an IPL tail with IPL index $\epsilon_{B}$ related to *H* by

(18) $$\epsilon_{B}=2-2H.$$

In summary, the experimental signal ξ(t), which is the object of our statistical analysis, generates the stationary correlation function

(19) $$\Phi_{\xi }\left(t\right)={\left(\frac{T}{T+t}\right)}^{\epsilon_{B}},$$

where $\epsilon_{B}\ll 1$ when *H* is very close to the maximal value of 1. According to the Wiener-Khinchine theorem [10], the power spectrum is the Fourier transform of the auto-correlation function:

(20) $$S\left(\omega \right)=\frac{1}{2\pi }\int_{-\infty }^{+\infty }dtexp\left(i\omega t\right)\Phi_{\xi }\left(t\right).$$

Inserting the correlation function from Equation (19) into Equation (20) we obtain

(21) $$S\left(\omega \right)\propto \frac{1}{\omega^{\beta }},$$

where

(22) $$\beta =1-\epsilon_{B}.$$

We see that the ideal 1/f noise is obtained by setting the condition $\epsilon_{B}\to 0$, forcing *H* to be very close to the maximum value of 1. Using Equation (18) we obtain the widely used relation

(23) β=2H−1.

### 2.2. Aging Fractional Brownian Motion

In the case when the process is driven by crucial events with an IPL index in the range 2<μ<3, in the long-time limit a stationary correlation function identical to that of Equation (19) is generated. To avoid confusion, let us adopt the following notation

(24) $$\Phi_{\xi }\left(t\right)={\left(\frac{T}{T+t}\right)}^{\epsilon_{C}},$$

and not Equation (19) since we have used the IPL for crucial events and not Equation (17). Please note that in this case, due to Equation (14),

(25) $$\epsilon_{C}=\mu -2.$$

We understand the temptation to assume that $\epsilon_{C}=\epsilon_{B}$, yielding

(26) $$H=\frac{4-\mu }{2}.$$

This relation is, however, not correct because, as proven elsewhere, crucial events generate [5] the scaling index

(27) $$\eta =\frac{1}{\mu -1}.$$

Please note that when the fluctuation ξ(t) hosts crucial events whose power spectrum has the IPL index:

(28) β=3−μ,

this equation is valid throughout the whole range 1<μ<3. In the case μ>2, Equation (28) is recovered by using Equation (22) with $\epsilon_{B}=\epsilon_{C}=\mu -2$.

The entropic analysis proposed by this paper, is a significant refinement of the technique of Diffusion Entropy Analysis (DEA) proposed in 2001, [11,12]. The DEA of Reference [11] was proposed as method of scaling evaluation based on observations of the experimental signal with no focus on the detection of crucial events. The DEA of [12] was proposed for the explicit purpose of detecting the scaling generated by crucial events, and was applied to surrogate sequences of crucial events with the explicit rule of forcing the random walker to make a jump ahead with the occurrence of an event. The refined form of DEA proposed in this paper is based on the DEA of [12]. In the case where we must analyze an experimental fluctuation and we do not use the method of stripes, the DEA of [12] is equivalent to the DEA of Reference [11]. Using the method of stripes, we define events, which may be either crucial or not, and we use the DEA of [12] to assess the fraction of crucial events imbedded in the set of events generated by the stripes. We shall see that this procedure affords a method to assess whether 1/f noise does host crucial events, or does not.

## 3. Entropy Concept

The concept of entropy has a thermodynamic origin and is closely connected to the second law of thermodynamics. The well-known expression [1]

(29) S=klnW

can be fruitfully used to explain why the free expansion of a gas in a container is an irreversible process [13]. However, the attempts to describe the time evolution towards equilibrium resting on the Gibbs entropy forced investigators to adopt the concept of a Gibbs ensemble average and, consequently, to assume ergodicity [14], namely that an average over infinitely many copies of the same system is identical to averaging a single system of the ensemble over time. At the same time, these attempts led the investigators to assume that chaos is an important ingredient to generate an irreversible transition to equilibrium, even if chaos may not be completely random [15]. The main problem of reconciling the second law of thermodynamics and irreversibility with the reversible nature of both classical and quantum mechanics led the investigators to establish a connection between the second law and information theory [16], as explained in the work of Landauer [17] and in the more recent work of Reference [18]. This reconciliation attempt becomes even harder when we move from quantum physics to the second law insofar as it raises the still unresolved problem of deriving classical from quantum physics [19]. These contributions to the field of non-equilibrium statistical physics, although generating fruitful applications, are based on the Gibbs ensemble perspective and consequently do not shed light into the dynamics of the individual systems of a statistical ensemble, if no recourse is done to the ergodicity assumption.

In the last ten years, however, increasing attention has been devoted to the ergodicity breaking, in two different fields of research. The first field of investigation is molecular diffusion, with the tracking of single molecules in living cells [5], making it possible to do time averages over the motion of a single molecule, and the latter is the field of complex networks, where the discovery of cooperation-induced criticality has proven to yield temporal complexity, namely non-ergodic fluctuations of the complex network’s mean field [20]. A reasonable conjecture currently being made is that in both cases non-ergodic behavior is a signature of the transition from a non-cooperative to a self-organized state [21]. The brain is an example of a complex system of this kind and its non-ergodic nature raises the important question of how to measure its complexity using time averages, or, equivalently, how to define the entropy of a single trajectory. We therefore explore the important issue of Kolmogorov complexity [22], which is expected to shed light into the entropy of a single time series and thus on the entropy of a single trajectory, which is called Kolmogorov-Sinai (KS) entropy. We discuss the joint use of compression and diffusion [23], two methods of analyzing of time series based on the KS entropy. While the former procedure establishes the amount of order by the numerical evaluation of algorithmic compressibility of a time series, the latter assesses the amount of order by forcing the time series to generate diffusion, the scaling of which is sensitive to the deviation from randomness.

We assume that complexity is generated by the occurrence of renewal non-Poisson processes, and on the basis of this assumption we propose an approach to calculating the entropy of a single trajectory based on the theoretical perspective of a continuous random walk [24]. Randomness is a property of events occurring in the operational time ψ, related to the clock time *t* by the relation $\psi =t^{\alpha }$. This observation leads to a generalization of the Pesin’s identity [25] that we propose to adopt to define the entropic complexity of non-ergodic trajectories. We conclude this section arguing that non-ergodic fluctuations may be incompressible despite their vanishing Lyapunov exponent and we discuss to what extent this theoretical perspective may afford a useful technique of analysis of non-stationary time series.

### 3.1. External Entropy

The authors of [26] shed light on the paradoxical macroscopic effects generated by chaotic trajectories exploring regions with different Lyapunov coefficients [15] used the concept of external entropy that was later adopted in Reference [23] to discuss the Kolmogorov complexity in terms of compression and diffusion. To evaluate external entropy for non-ergodic trajectories, we can benefit from the time series generated by the idealized Manneville map [23]. Imagine a particle moving in the interval [0,1] from an initial condition 1>y>0, with uniform probability, according to the dynamical prescription

(30) $$\dot{y}=\lambda y^{z},$$

with z>1 and λ<<1. The time τ necessary to arrive at the border y=1 moving from an initial value 0<y<1 is given by

(31) $$\tau =\frac{\mu -1}{\lambda }\left\{\frac{1}{y^{\frac{1}{\mu -1}}}-1\right\},$$

with

(32) $$\mu \equiv \frac{z}{z-1}.$$

When the particle reaches the border y=1 it is injected back to a new initial position by randomly selecting a new number *y*. It is important to notice that the waiting time probability distribution function (PDF) ψ(τ) separating consecutive activations of the external entropy has the same analytical form as the waiting time PDF ψ(τ) of Equation (10). Notice that when μ>2, we recover the mean waiting time given by Equation (11) with

(33) $$T=\frac{\mu -1}{\lambda }.$$

Please note that the limiting case μ=∞ is realized by replacing the procedure of Equation (31) with

(34) $$\tau =\Gamma ln\left(\frac{1}{y}\right),$$

which is easily shown to generate the Poisson waiting time PDF ψ(τ)

(35) ψ(τ)=Γexp(−Γτ).

The main remaining question is whether DEA can properly address the condition μ<2, which is characterized by perennial ergodicity breaking.

The authors of Reference [27] proved that the Kolmogorov-Sinai entropy, $\eta_{KS}$, reads:

(36) $$\eta_{KS}=z\left(2-z\right)ln2,$$

where, according to Equation (32),

(37) $$z\equiv \frac{\mu }{\mu -1}.$$

The land of crucial events ranges from z=1.5,μ=1.5, to z=∞,μ=1, with $\eta_{KS}$ vanishing from z=2 to z=∞. We can recover the proposal of Korabel and Barkai [25] by the following conjectures on the computational cost. The computation cost, in the case μ>2 increases linearly in time, while in the case μ<2 it increases as $t^{\alpha }$, with

(38) α=μ−1,

In fact, the number of random drawings *n* of the initial condition *y* is

(39) $$n\propto \frac{t}{<\tau >}$$

for μ>2. This is the condition making it possible to realize a Lévy walk [28,29]. In the case μ<2, as earlier mentioned, the number of random drawings is

(40) $$n\propto t^{\alpha }.$$

This simple heuristic prediction has the effect of defining the incompressibility of the time series also for μ<2 [25]. In fact, adopting the generalized form of KS entropy proposed by Korabel and Barkai [25], based on observing that for μ<2, it is necessary to use a new definition of time, taking into account the transition from *n* proportional to *t* to *n* proportional to $t^{\alpha }$, a singularity appears at z=2. The generalized $h_{KS}$ of Korabel and Barkai [25] is a decreasing function of *z* for z<2 and it becomes an increasing function of *z* for z>2. These intuitive arguments can be used to attract attention to the rigorous work of References [30,31,32,33,34]. Here we limit ourselves to point out that informational compressibility implies the existence of a message carried by the time series, thereby implying a connection with cognition.

We emphasize that according to the statistical analysis of the brain in the awake condition [35] the brain is controlled by an AFBM process with μ=2. This means that the brain operates at the border between the perennial aging condition, μ<2, and the temporary lack of stationarity condition, μ>2. The observation of 1/f noise does not make it possible to realize the singularity of this condition because the IPL index β of the spectrum obeys Equation (28), making the spectrum become that of an ideal 1/f noise at μ=2, with β moving in a continuous way from values slightly larger than 1 to values slightly smaller. The singularity of this condition, made evident by the entropic analysis of Korabel and Barkai, should be properly taken into account by the analysis of real physiological data.

### 3.2. Entropic Treatment of the Scale Detection Issue

The existence of a message with a meaning motivates us to move from the Boltzmann to the Wiener/Shannon entropy. Let us imagine that we have a sequence of events and that all of them are crucial. We follow the prescription of Reference [12]. For any crucial event, the random walker makes a step ahead by the fixed quantity 1 and thereby builds up a diffusion trajectory x(t). We then observe this trajectory with the moving window of size *l* so as to generate the histogram of the PDF p(x,l). According to the generalized central limit theorem [12] we obtain

(41) $$p\left(x,l\right)=\frac{1}{t^{\eta }}F\left(\frac{x}{l^{\eta }}\right),$$

where the power law index is

(42) η=μ−1,

if 1<μ<2, and becomes

(43) $$\eta =\frac{1}{\mu -1},$$

if 2<μ<3. Finally, we have

(44) η=0.5,

if μ>3.

It is important to stress that these rules in the anomalous case generate the asymmetric Lévy diffusion [12].

To appreciate the main result of this paper, namely, how a refined version of DEA makes it possible to distinguish AFBM from SFBM, it is convenient to discuss DEA in action on an experimental signal ξ(t), when we do not know if the single fluctuations are crucial or not crucial. Let us consider, for instance, the case when the laminar regions between two consecutive crucial events are filled with either 1’s or −1’s, with a coin tossing prescription, and 2<μ<3. This is the celebrated Lévy walk [28,29]. We create the diffusion trajectory

(45) $$x\left(t\right)=\int_{0}^{t}dt^{\prime }\xi \left(t^{\prime }\right).$$

We then observe this trajectory with the mobile window of size *l*. We evaluate the difference between the value of x(t) at the end of the window and the value of x(t) at the beginning of the window. This allows us to create the probability density function

(46) $$p\left(x,l\right)=\frac{1}{l^{\eta }}F\left(\frac{x}{l^{\eta }}\right).$$

In this case, the scaling η is identical to that of the asymmetric Lévy scaling of Equation (43).

Let us also discuss the case where the signal does not host any crucial event. If ξ(t) is a generator of FBM the observation of the trajectory of Equation (45) with the method of the mobile window of length *l* yields the probability density function

(47) $$p\left(x,l\right)=\frac{1}{l^{H}}F\left(\frac{x}{l^{H}}\right).$$

The main problem with the use of the mobile window of size *l* [2] is that in the case of crucial events, Equation (41), the long, slow tails of the PDF make their second moment divergent, and the scaling evaluation is affected by the numerical truncation that cannot be easily controlled. The second moment technique works in the case of SFBM because in this case the PDFs are Gaussian, with fast exponential tails.

This difference in the second moment is the reason the adoption of DEA [11,12] turns to be very convenient. In fact, DEA lead us to evaluate the Wiener/Shannon entropy of the diffusion process, namely

(48) $$S\left(l\right)=-\int_{-\infty }^{+\infty }p\left(x,l\right)lnp\left(x,l\right)dx.$$

Inserting Equation (41) into Equation (48) yields

(49) S(l)=A+ηlnl,

where A is a constant. When we insert Equation (47) into Equation (48) and integrate, we obtain

(50) S(l)=B+Hlnl,

where *B* is a different constant from A. The scaling parameter η is properly defined even if the probability density function of Equation (41) has a diverging second moment. The DEA does perceive the correct scaling η even if the long-time limit leads to numerical statistical inaccuracy, since the crucial events are rare in this limit. However, this use of DEA does not allow us to assess if we are dealing with a SFBM or an AFBM phenomenon. In fact, if η>0.5 it is impossible to establish with the use of DEA alone if we are observing super-diffusion generated by SFBM or by AFBM.

## 4. Refined Diffusion Entropy Analysis

This section is devoted to proving that DEA becomes an efficient detector of crucial events when it is supplemented by the method of stripes and we compare its results to those obtained without using stripes.

### 4.1. Without Stripes

Here we explain why DEA detects crucial events even when the ordinary methods fail. We need to explain why crucial events are invisible to the ordinary methods of statistical analysis. To accomplish this, we create a time series where the crucial events are imbedded in a dense set of Poisson events, and using the conventional method of correlation functions it turns out to be difficult to evaluate the complexity of crucial events. To prove this important property, we generate a surrogate sequence ξ(t) according to the prescription

(51) $$\xi \left(t\right)=\epsilon \xi_{C}\left(t\right)+\left(1-\epsilon \right)\xi_{P}\left(t\right).$$

**Figure 1:**
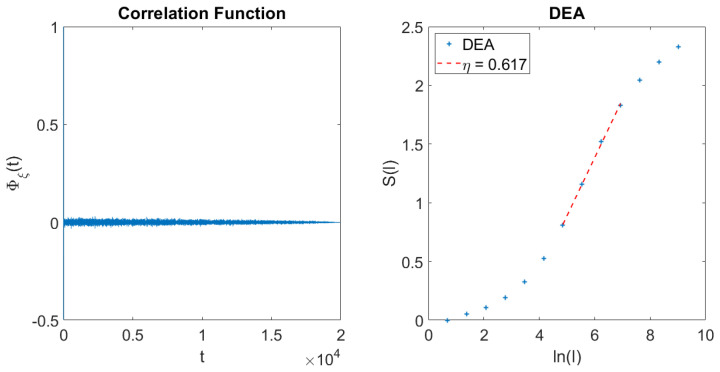
Effect of embedding crucial events in a dense cloud of Poisson events. **Left panel:** Correlation function of the time series of Equation (51), ϵ=0.1, Γ=1. λ=1.6, μ=2.6; **Right panel:** DEA without stripes. The slope of the intermediate asymptotics fits the prediction of Equation (43).

The fluctuation $\xi_{C}\left(t\right)$ is obtained by generating the crucial events using Equation (31) and filling the laminar regions with either 1 or −1 using a coin tossing procedure. The fluctuation $\xi_{P}\left(t\right)$ is obtained by generating the crucial events using Equation (34) and filling again the laminar regions with either 1 or −1 using a coin tossing procedure. We are studying the case 2<μ<3, namely the Lévy walk case, which is characterized in the long-time limit by a stationary correlation function. The equilibrium correlation function of the fluctuation ξ(t) is expected to be given by

(52) $$\Phi_{\xi }\left(\tau \right)=\frac{{\left(1-\epsilon \right)}^{2}exp\left(-\Gamma \tau \right)+\epsilon^{2}{\left(\frac{T}{\tau +T}\right)}^{\mu -1}}{\epsilon^{2}{\left(1-\epsilon \right)}^{2}},$$

where the second term is the equilibrium correlation function of the signal generated by crucial events. In the case where ϵ≪1 the crucial events generate a weak tail that can be mistaken for noise. In fact, the left panel of Figure 1 shows that after the fast decay due to the Poisson events a weak tail appears. This tail contains information about crucial events, but it is virtually impossible to derive the crucial index 2<μ<3 from it. The adoption of DEA without using the method of stripes, on the contrary, makes it possible to derive with sufficient accuracy the value of μ using

(53) $$\mu =1+\frac{1}{\eta },$$

which is illustrated by the panel on the right of Figure 1. The reason for this important property is that DEA determines the scaling in the time asymptotic limit. The scaling generated by the Poisson events is η=0.5. The scaling generated by the crucial events for μ>2 is given by Equation (43). The adoption of the coin tossing prescription for the extended laminar region has the effect of generating a Lévy walk [36]. This is so because in this case we are allowed to use Equation (39) with the Lévy walk generating super-diffusion. It is surprising that despite many changes of sign due to the high density of Poisson events the DEA technique, without the use of stripes, in the long-time region is sensitive only to crucial events. The intuitive explanation is that the crucial events, yielding a diffusion scaling η>0.5, despite their low density generate a broader distribution density and therefore a predominant contribution to the scaling parameter η.

### 4.2. With Stripes

In this section, we show that the joint use of DEA with the method of stripes and without the method of stripes makes it possible for us to establish if super-diffusion is generated by SFBM or AFFBM. Let us denote with the symbol ξ(t) the experimental time series under study. Rather than converting it into the diffusion process x(t) directly, we follow the method adopted by Allegrini et al., 2002 [37]. We divide the ordinate axis into bins of size *s* and record the times at which the experimental signal crosses the axis. In this way we obtain a time series $\left\{t_{i}\right\}$. At any of these times an event occurs. We do not know if this event is crucial or not. We replace the experimental fluctuation ξ(t) with a time series z(t) defined as follows. If time *t* coincides with one of the times $t_{i}$, we set z(t)=1 and z(t)=0 otherwise. In other words, if the time *t* corresponds to the occurrence of an event, the random walker makes a step ahead by the fixed quantity 1. The diffusion trajectory x(t) is obtained from

(54) $$x\left(t\right)=\int_{0}^{t}dt^{\prime }z\left(t^{\prime }\right).$$

**Figure 2:**
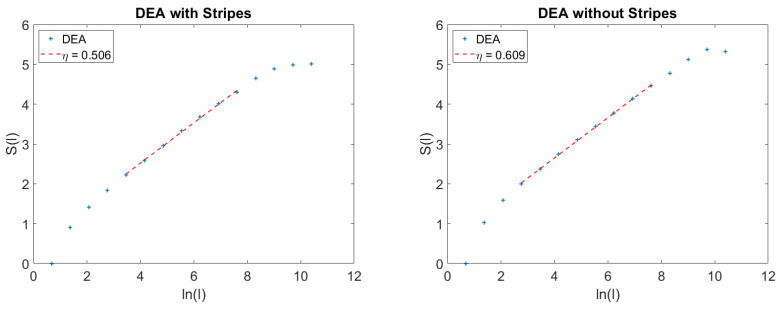
Comparison between the use of DEA with stripes, left panel, and the use of DEA without stripes, right panel.

Figure 2 refers to the case where H=0.6, namely, the case of anomalous super-diffusion due to SFBM. The right-hand panel of Figure 2 shows that the ordinary DEA with no stripes yields η=H>0.5, thereby correctly recognizing super-diffusion. The left-hand panel of Figure 2 shows that the use of stripes yields η=0.5, thereby killing the contribution of SFBM to super-diffusion. This is the important property that we use to establish if the anomalous scaling is due to AFBM or to SFBM.

The theoretical reason for this result is connected to the recrossing of the origin of FBM ξ(t). According to Sinn [38] the time distance between consecutive origin crossings is described by the exponential waiting time survival probability

(55) $$\Psi \left(t\right)=e^{-tln\left(\frac{1}{\frac{2}{\pi }arcsin2^{H-1}}\right)}.$$

We make the plausible conjecture that the exponential structure is a general property of the FBM crossing. The method of stripes generated many events that can be approximately interpreted as Poisson. Of course, the crossing is characterized also by memory that generates a weak deviation from ordinary scaling.

What about embedding crucial events into a dense set of non-crucial events generated by a SFBM? To answer this question, we create the surrogate sequence

(56) $$\xi \left(t\right)=\epsilon \xi_{C}\left(t\right)+\left(1-\epsilon \right)\xi_{FBM}\left(t\right),$$

where $\xi_{FBM}\left(t\right)$ is generated according to the Mandelbrot algorithm of Reference [39]. In this case, it is necessary to exorcise the misleading influence of SFBM and we must use DEA with the method of stripes, which is proved by Figure 2 to kill the anomalous scaling generated by SFBM. In this case, rather than filling the laminar regions between two crucial events with either 1 or −1, as done for Figure 1 and Figure 2, we adopt a different method that will make it easier for us to use the detailed illustration of the method of stripes of Section 5, to show that this method is only sensitive to crucial events. When a crucial event occurs, we assign to it an intensity proportional to the time distance from the occurrence of earlier crucial events. To explain the effect of this procedure on the scaling evaluated using the ordinary method of ensemble average we must make an excursion to the earlier work of References [40,41]. The intensity of the fluctuations has a divergent second moment, thereby yielding the diffusion scaling

(57) $$\eta =\frac{1}{\mu -1}.$$

However, the method of stripes depends only on the statistics of crucial events and it is independent of the anomalous intensity of the crucial events as well as of SFBM.

**Figure 3:**
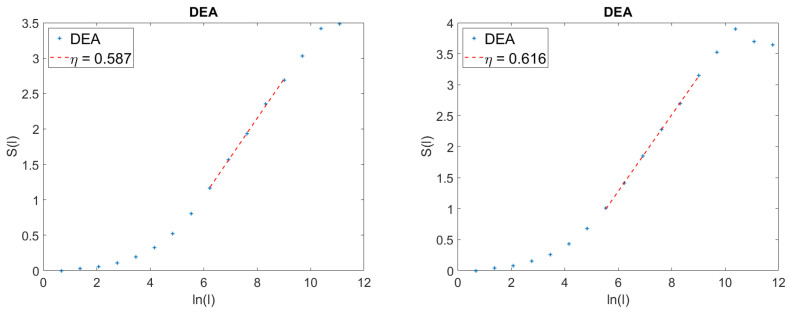
Effect of embedding crucial events in a dense cloud of Poisson events compared to the effect of embedding them into a cloud of SFBM fluctuations. In both panels ϵ=0.1, λ=1.6, μ=2.6; **Left panel:** Γ=1. **Right panel:** H=0.7.

All this is described by Figure 3, where the left panel refers to the time series of Equation (51) and the right panel to the time series of Equation (56). We see that the stripes kill the anomalous SFBM scaling, H=0.7, which is larger than the AFBM scaling, η=0.625. It is interesting to notice that the density of non-crucial events in both figures is the same, and the scaling detected by DEA with stripes is in both cases the scaling generated by crucial events. In other words, the method of stripes makes the AFBM fluctuations equivalent to Poisson fluctuations.

Finally, we detect the time distances $\tau_{i}$ between two consecutive crossings of the origin for both the case (51) and (56). We evaluate the cross-correlation function

(58) $$C_{ij}=\frac{(\langle \tau_{i}-\bar{\tau })\left(\tau_{j}-\bar{\tau }\right)\rangle }{\langle \Delta \tau^{2}\rangle -{\langle \Delta \tau \rangle }^{2}}.$$

**Figure 4:**
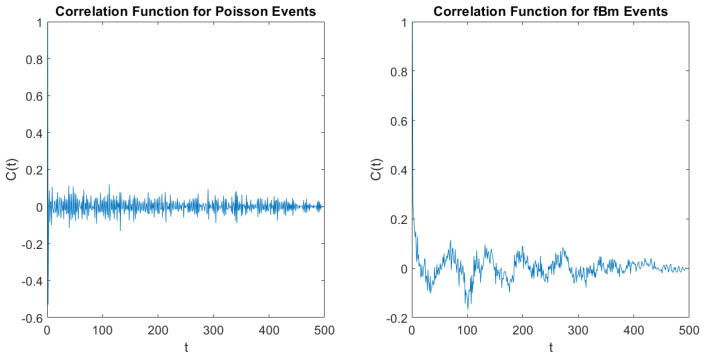
Correlation function $C_{ij}$ of Equation (58). Left panel: $C_{ij}$ of the signal ξ(t) of Equation (51).

This correlation function is stationary, namely, $C_{ij}=C\left(t\right)$, with t≡|i−j|. In the scale of Figure 4 the integer variable *t* is virtually equivalent to a continuous variable. Figure 4 shows that when the non-crucial events are a form of SFBM the tail of C(t) reveals the existence of a SFBM process to the time series under study. The analysis of heartbeats of References [37,42] in addition to finding the correct crucial scaling of heartbeats, corresponding to the parameter μ slightly larger than 2, with the method of stripes, yields for C(t) the slow tail that is a signature of SFBM.

## 5. Details on the Action of the Stripes

**Figure 5:**
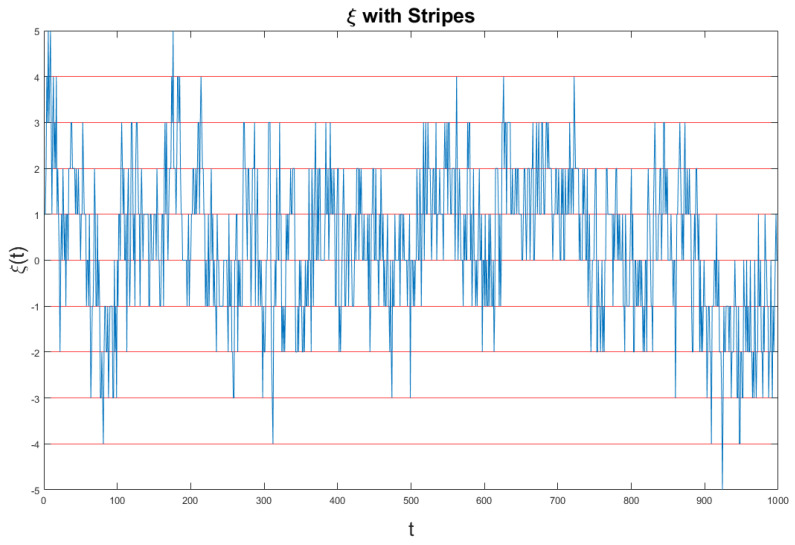
This figure illustrates the signal ξ(t) used as surrogate sequence for the method of DEA illustrated in Figure 3.

We devote this Section to more details on the method of the stripes. We afford details in the case of the results of Figure 3. This is expected to help the readers to also get a better understanding of the method used to get the results of the left panel of Figure 2. In Figure 5 we show the fluctuation ξ(t) generated by Equation (56), which is the combination of crucial and non-crucial events adopted by us the generate the diffusional trajectory of Equation (54).

The ordinate axis is divided in many stripes of size s=1. The time at which the signal ξ(t) moves from one stripe to one of the two nearest neighbor stripes is recorded as the time of occurrence of an event, which can be either crucial, if it depends on $\xi_{C}\left(t\right)$, or not, if it depends on $\xi_{FBM}\left(t\right)$. In the case of fluctuations of intensity much larger than the size *s* of the stripes many events occur at the same time and they are recorded as a single event. This explains why the large intensities of the crucial fluctuations do not influence the scaling, but only their temporal occurrence does.

The adoption of the always stepping ahead rule [12] to define the diffusional trajectory x(t) of Equation (54) is essential to making the method of stripes efficient. In fact, if all the events are crucial, this diffusion process generates the scaling η=1/(μ−1), which is larger than 0.5 for μ<3. If all the events are not crucial, η=05. When the events detected using the stripes are a mixture of crucial and non-crucial events, the long-time limit of the diffusion process is dominated by the faster scaling of crucial events. This explains why the adoption of the method of stripes makes the DEA so efficient for the detection of crucial events.

**Figure 6:**
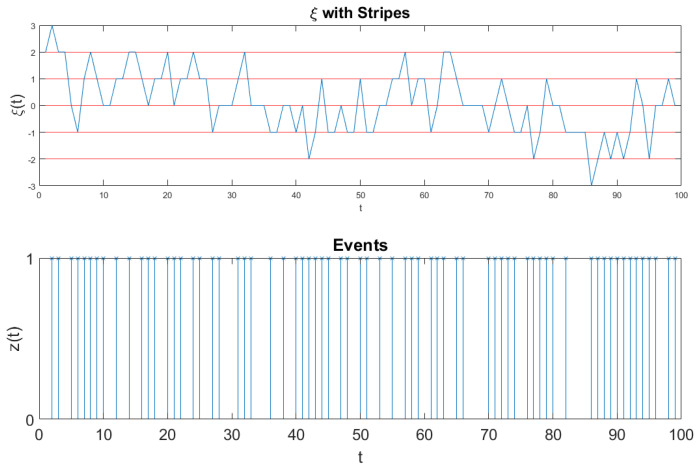
The top panel illustrates the fluctuation ξ(t) of Equation (56). The bottom illustrates the jumps z(t) done by the random walker to generate the trajectory x(t) of Equation (54). The random walker makes a jump ahead of intensity 1 when the fluctuation ξ(t) of the top panel crosses the border lines between consecutive stripes.

The fluctuation ξ(t) of Figure 5 is too dense to see the single events. Therefore, we invite the readers to look at Figure 6. Also Figure 6 refers to the analysis of the surrogate sequence of Equation (56) and to the results of Figure 3. However, the realization ξ(t) of Figure 6, is of size 100, much shorter than the realization of ξ(t) of Figure 5: it is short enough as to illustrate the details of this fluctuation. The times at which the signal ξ(t) crosses one of the border lines are recorded to signal the occurrence of an event, which can be either crucial or not, according to the component of ξ of Equation (56), either $\xi_{C}\left(t\right)$ or $\xi_{FBM}$, determining the crossing. We convert these events into the diffusional trajectory x(t). This is shown in detail in Figure 6.

## 6. Criticality and Physiological Processes

Self-organized criticality (SOC) and the connection between SOC and 1/f noise have been the subject of sometimes heated debates since the publication of the original work of Bak and coworkers [8]. The connection between criticality and 1/f noise has been emphasized [43,44] and questioned [45,46], and it remains an open question especially because whether or not 1/f noise itself has a unique origin is still unsettled.

On one side we have proposals that advocate to some extent the adoption of SFBM arguments [47,48] and introduce the adoption of multifractality to take into account the fluctuations of *H*. On the other side there are approaches to 1/f noise in systems with ergodicity breaking [49] including the extreme case of perennial aging [50] with μ<2. The analysis of the brain dynamics of Reference [35] led these authors to the conclusion that the brain in the awake state is a generator of an ideal 1/f noise, corresponding to μ=2.

Another important physiological process is the dynamics of the heart and significant interest exists on the correlation between heart rate variability and brain activity [51,52,53]. However, the theoretical explanation of the correlation between these two important physiological processes remains a difficult problem due to their different frequency scales.

The main theoretical result of the present paper allows us to reach some compelling conclusions about the nature of these two physiological processes. In fact, the heartbeats have been analyzed with the method of the stripes [37,42] leading the conclusion that the IPL index μ in the case of healthy individuals is close to the condition generating ideal noise, μ=2. In a more recent paper, Bohara et al. [54] using the method of the stripes analyzed the EEGs of healthy subjects in the same awake conditions as the human subjects of Reference [35] and confirmed that they have a power law index μ very close to the ideal condition μ=2. They also established a bridge between waves and crucial events leading to the important conjecture that for the brain-heart correlation the tuning of frequencies is not as important as the tuning of temporal complexity, namely the brain and heart dynamics sharing the same IPL index μ.

On the basis of the present results, we conclude that the frequencies of the brain wave may affect the brain physiological process with SFBM contributions that are killed by the DEA analysis resting on the adoption of the method of stripes and that the form of 1/f noise generated by the brain in the awake condition is due to ASBM, thereby supporting the conjecture that the brain-heart correlation is a consequence of complexity matching [55], mainly resting on tuning temporal complexity rather than frequencies.

It is important to stress that the theoretical perspective of this paper affords strong support to our conviction that crucial events play a fundamental role for cognition, without ruling out coherence. The authors of the recent work of Reference [56] found that meditation has the remarkable role of enhancing coherence. We believe that SFBM is closely related to coherence. On the other hand, the earlier work of Reference [37], where the method of stripes has been used for the first time, suggests that crucial events are imbedded in a cloud of Poisson events, ruling out coherence-induced SFBM. This paper shows that the crucial events may be imbedded in a cloud of SFBM fluctuations and that also in this case, thanks to the method of stripes, the action of crucial events can be revealed. The right panel of Figure 4 illustrates SFBM effects that the DEA with stripes does not perceive.

## 7. Concluding Remarks

We believe with the authors of the recent paper [57] that 1/f noise is a manifestation of Self-Organized Temporal Criticality (SOTC) [58], a new form of self-organization generating crucial events and, consequently, the pink noise generated by an AFBM process. There is general agreement that the emergence of life is signaled by a departure from the ordinary scaling η=0.5 [2]. Whether this departure from ordinary statistical physics rests on SFBM, or AFBM, or a joint action of both processes is still a subject of research and possible debate. The DEA approach using the method of stripes illustrated in this paper will contribute to progress in this field of research, which is not limited to applications in biological and physiological processes. The discussion on the origin of cognition moves from the elementary biological process, for instance cell to cell communication [59,60,61], generating the conjecture that this is based on quantum mechanical coherence, one form of non-commutative probability theory, a theoretical perspective that we believe to be related to SFBM processes on the basis of the fact that the dynamical approach to FBM [4] was derived by the quantum mechanical theory of quantum dissipative processes of Weiss [62]. We think that at this elementary biological level crucial events, namely form of compressible randomness, are generated by SOTC. This fundamental problem goes far beyond elementary biological processes. In fact, the fast and slow thinking aspects of decision-making processes [63,64] establish a complex interaction [65] between the brain of the single individuals and the society networks.

## Abbreviations

The following abbreviations are used in this manuscript:

FBM	Fractional Brownian Motion
SFBM	Stationary Fractional Brownian Motion
AFBM	Aging Fractional Brownian Motion
DEA	Diffusion Entropy Analysis
PDF	Probability Distribution Function
IPL	Inverse Power Law
SOTC	Self-Organized Temporal Criticality
EEG	Electroencephalogram
